# Interval estimation of the overall treatment effect in a meta-analysis of a few small studies with zero events

**DOI:** 10.1016/j.conctc.2017.11.012

**Published:** 2018-01-09

**Authors:** Konstantinos Pateras, Stavros Nikolakopoulos, Dimitris Mavridis, Kit C.B. Roes

**Affiliations:** a Department of Biostatistics and Research Support, Julius Center for Health Sciences and Primary Care, University Medical Center Utrecht, 3508 GA Utrecht, The Netherlands; b Department of Primary Education, School of Medicine, University of Ioannina, University Campus, 45110 Ioannina, Greece; c Department of Hygiene and Epidemiology, School of Medicine, University of Ioannina, University Campus, 45110 Ioannina, Greece

**Keywords:** Meta-analysis, Zero events, Small populations, Rare diseases, Heterogeneity

## Abstract

When a meta-analysis consists of a few small trials that report zero events, accounting for heterogeneity in the (interval) estimation of the overall effect is challenging. Typically, we predefine meta-analytical methods to be employed. In practice, data poses restrictions that lead to deviations from the pre-planned analysis, such as the presence of zero events in at least one study arm. We aim to explore heterogeneity estimators behaviour in estimating the overall effect across different levels of sparsity of events. We performed a simulation study that consists of two evaluations. We considered an overall comparison of estimators unconditional on the number of observed zero cells and an additional one by conditioning on the number of observed zero cells. Estimators that performed modestly robust when (interval) estimating the overall treatment effect across a range of heterogeneity assumptions were the Sidik-Jonkman, Hartung-Makambi and improved Paul-Mandel. The relative performance of estimators did not materially differ between making a predefined or data-driven choice. Our investigations confirmed that heterogeneity in such settings cannot be estimated reliably. Estimators whose performance depends strongly on the presence of heterogeneity should be avoided. The choice of estimator does not need to depend on whether or not zero cells are observed.

## 1 Introduction

Meta-analyses (MAs) techniques are commonly employed in order to obtain a more precise and more general effect estimate of a treatment. Heterogeneity (*τ*) of treatment effects measured in multiple Randomized Controlled Trials (RCTs) is a crucial part of the estimation [1].

In MAs of RCTs, methodological challenges arise when the disease under examination is rare and only a few small RCTs are available [2,3]. This is mostly due to the large sample assumptions on which most MA methods are based. In the case of rare diseases with binary endpoints, zero cells are more likely to be observed in at least one of the treatment arms of at least one contributing trial [[4], [5], [6]]. Zero cells in MAs pose challenges as they induce bias in both the estimation of the overall effect and the between-study variance (heterogeneity) [[7], [8], [9], [10], [11], [12], [13], [14]].

When conducting a MA, the estimation method might be adjusted conditionally on observing zero cells. Corrections are typically introduced by adding a number to the zero cells observed; furthermore, the choice of the heterogeneity estimator could change. The latter choice is by itself a challenging task, given the large pool of options [[15], [16], [17], [18], [19], [20], [21], [22], [23], [24]]. Prospective choice of analysis strategies is a fundamental element of statistical inference. The extent to which conditional (on the observed zero cells) analysis choices can affect robustness is of obvious concern.

Especially for dealing with a MA of a few RCTs, there is no straightforward answer to which estimator would be robust across several heterogeneity assumptions [21]. Most estimators face difficulties in case of a limited number of trials; they induce bias in the estimation of *τ* [25,26] and may result in inappropriate interval estimation of the treatment effect. However, not much is known regarding their behaviour in the presence of zero cells and small populations.

The primary objective of this work is to assess the robustness of heterogeneity estimators in the (interval) estimation of treatment effect across ranges of sparsity of events and assumed heterogeneity. The starting point is the acknowledged poor estimation of heterogeneity in this setting. We evaluate the estimators in case they are predefined (unconditional), as well as when they are chosen depending on the observed zero cells in contributing trials (conditional on the observed data, in short: conditional), and explore whether such a retrospective analysis choice can substantially affect inference.

The paper is organized as follows. First we describe the standard random-effects (RE) model and introduce the heterogeneity estimators briefly. Subsequently, we present two motivating examples and their analysis. Then we describe the simulation study and evaluate the two distinct approaches. We conclude with recommendations on evidence synthesis for a sparse-events MA in small populations.

## 2 Methods

We consider a set of *k* trials with binary outcomes that compare an experimental treatment to a control. Patients are randomized between two groups: treatment (T) and control (C).

By $Y_{i}$ we denote the log odds ratio (logOR) in the $i^{th}$ trial. Following standard theory (e.g. Ref. [1]), we assume:

(1) $$Y_{i}|\theta_{i}∼N\left(\theta_{i},\sigma_{i}^{2}\right),\;i=1,\ldots ,k$$

The study-specific treatment effect estimates are ${\hat{\theta }}_{i}=log\left(\frac{r_{Ti}\cdot \left(n_{Ci}-r_{Ci}\right)}{r_{Ci}\cdot \left(n_{Ti}-r_{Ti}\right)}\right)$, while their variances are $s_{i}^{2}=\frac{1}{r_{Ti}}+\frac{1}{n_{Ti}-r_{Ti}}+\frac{1}{r_{Ci}}+\frac{1}{n_{Ci}-r_{Ci}}$, where $r_{i}$ and $n_{i}$ denote the number of responders and the total number of subjects in each trial, respectively.

Assuming a fixed-effects (FE) model, *θ* is common for all studies ($\theta_{i}=\theta$). Assuming a RE model, the $\theta_{i}$ are considered exchangeable and follow a normal distribution, that is,

(2) $$\theta_{i}|\theta ,\tau^{2}∼N\left(\theta ,\tau^{2}\right)$$

where *θ* is the overall effect and $\tau^{2}$ is the between-study variance. When $\tau^{2}=0$, then the RE model reduces to the FE model. The pooled effect estimate is calculated as a weighted average $\hat{\theta }=\sum_{i}w_{i}Y_{i}/\sum_{i}w_{i}$. The inverse variance (IV) weights are then defined as $w_{i,RE}=1/\left(s_{i}^{2}+{\hat{\tau }}^{2}\right)$ for the RE model and as $w_{i,FE}=1/s_{i}^{2}$ for the FE model.

A standard confidence interval is calculated as, $\hat{\theta }\pm {\hat{\sigma }}_{\theta }\;z_{1-a/2}$, where $z_{1-a/2}$ is the (1−a/2) quantile of the standard normal distribution and ${\hat{\sigma }}_{\theta }=\sqrt{1/\sum_{i}w_{i}}$.

To apply the RE model, estimation of heterogeneity is required. In the presence of zero cells, heterogeneity estimators entail the addition of a small continuity correction (CC) on zero cells in order to provide finite estimates. Several methods for estimating $\tau^{2}$ are proposed in the literature. Table 1 presents a summary of the 15 estimators that are included in this study. For a detailed overview of heterogeneity estimators, we refer the reader to two systematic reviews [27,28].

**Table 1:** Summary of heterogeneity estimators, including their equation, abbreviation and source.

Methods	Equation	Abbreviation	Source
DerSimonian Laird	${\hat{\tau }}_{dl}^{2}=max\left(0,\left(Q_{FE}-\left(k-1\right)\right)/c_{FE}\right)$	dl	[15]
Positive DerSimonian Laird	${\hat{\tau }}_{dl_{p}}^{2}={\hat{\tau }}_{dl}^{2},{\hat{\tau }}_{dl}^{2}>0$ and ${\hat{\tau }}_{dl}^{2}=0.01,{\hat{\tau }}_{dl}^{2}<=0$	dlp	[17]
Two-step Der Simonian Laird	${\hat{\tau }}_{dl2}^{2}=max\left(0,Q_{RE}-\left(w_{i,RE}^{2}\;s_{i}^{2}-\frac{\sum_{i}w_{i,RE}^{2}\;s_{i}^{2}}{\sum_{i}w_{i,RE}}\right)/c_{RE}\right)$	dl2	[16]
Hedges	${\hat{\tau }}_{he}^{2}=max(0,\frac{\sum_{i}{\left(Y_{i}-{\bar{Y}}_{FE}\right)}^{2}}{k-1}-\frac{\sum_{i}s_{i}^{2}}{k})$	he	[24]
Two step Hedges	Similar to DL2 using the Hedges estimator	he2	[16]
Positive Sidik-Jonkman	${\hat{\tau }}_{sj}^{2}=max\left(\frac{\sum_{i}\left({\left(Y_{i}-\bar{Y_{FE}}\right)}^{2}/\left(r_{i}+1\right)\right)}{k-1},0.01\right)$, $r_{i}=s_{i}^{2}/{\hat{\tau }}_{O}^{2}$	sj	[20]
Model error variance - vc	${\hat{\tau }}_{mvvc}^{2}=\frac{\sum_{i}\left({\left(Y_{i}-\bar{Y_{FE}}\right)}^{2}//r_{i}^{*}+1\right)}{k-1}$, $r_{i}^{*}=s_{i}^{2}/{\hat{\tau }}_{HE}^{2}$	mvvc	[20]
Paul-Mandel	$\left(\tau_{pm}^{2}\right)$, $F\left(\tau^{2}\right)=\sum_{i}w_{i,RE}{\left[Y_{i}-Y_{w}\left(\tau^{2}\right)\right]}^{2}-\left(k-1\right)$	pm	[18]
Improved Paul-Mandel	$\left(\tau_{ipm}^{2}\right)$, $F\left(\tau^{2}\right)=\sum_{i}w_{i,RE}^{*}{\left[Y_{i}-Y_{w}\left(\tau^{2}\right)\right]}^{2}-\left(k-1\right)$	Ipm	[19]
Hartung - Makambi	${\hat{\tau }}_{hm}^{2}=\frac{Q_{FE}^{2}}{\left[2\left(k-1\right)+Q_{FE}\right]c_{FE}}$	hm	[22]
Hunter-Schmidt	${\hat{\tau }}_{hs}^{2}=max\left(0,\left(Q_{FE}-k\right)/\sum_{i}w_{i,FE}\right)$	hs	[23]
Maximum Likelihood	${\hat{\tau }}_{ml}^{2}=max(0,\sum_{i}w_{i,RE}^{2}({\left(Y_{i}-{\bar{Y}}_{ML}\right)}^{2}-s_{i}^{2})/\sum_{i}w_{i,RE}^{2})$	ml	–
Restricted Maximum likelihood	${\hat{\tau }}_{reml}^{2}=max(0,\frac{\sum_{i}w_{i,RE}^{2}({\left(Y_{i}-{\bar{Y}}_{ML}\right)}^{2}-s_{i}^{2})}{\sum_{i}w_{i,RE}^{2}}+\frac{1}{\sum_{i}w_{i,RE}})$	reml	–
Rukhin Bayes zero estimator	${\hat{\tau }}_{rb0}^{2}=\frac{\sum_{i}{\left(Y_{i}-{\bar{Y}}_{FE}\right)}^{2}}{k+1}-\frac{\sum_{i}\left(n_{i}-k\right)\left(k-1\right)\sum_{i}s_{i}^{2}}{k\left(k+1\right)\sum_{i}\left(n_{i}-k+2\right)}$	rb0	[21]
Rukhin Bayesian positive	${\hat{\tau }}_{rbp}^{2}=\sum_{i}{\left(Y_{i}-{\bar{Y}}_{FE}\right)}^{2}/\left(k+1\right)$	rbp	[21]

$w_{i,RE}=\frac{1}{\left(s_{i}^{2}+\tau^{2}\right)},w_{i,FE}=\frac{1}{s_{i}^{2}}$, ${\bar{Y}}_{RE/FE}=\frac{\sum_{i}w_{i,RE/FE}Y_{i}}{\sum_{i}w_{i,RE/FE}}$, $Q_{RE/FE}=\sum_{i}\;w_{i,RE/FE}{\left(Y_{i}-{\bar{Y}}_{RE/FE}\right)}^{2}$, $c_{RE/FE}=\sum_{i}\;w_{i,RE/FE}-\frac{\sum_{i}w_{i,RE/FE}^{2}}{\sum_{i}w_{i,RE/FE}}$, $w_{i}^{*}=\frac{1}{\left(\tau^{2}+v_{i,ipm}^{*}\right)},v_{i,iPM}=\frac{1}{n_{\left(T,i\right)}+1}\left(e^{-Prc_{O}-\tilde{Y}+\tau^{2}/2}+2+e^{Prc_{O}+\tilde{Y}+\tau^{2}/2}\right)+\frac{1}{n_{\left(Ci\right)}+1}\left(e^{-Prc_{O}}+2+e^{Prc_{O}}\right)$$Pr_{c,o}$: Observed control event rate, $\tau_{O}^{2}=\sum_{i}\left(Y_{i}-\bar{Y_{FE}}\right)/k$. The pm, ipm, ml and reml are iterative estimators.

## 3 Motivating examples

### 3.1 Intravenous immunoglobulin (IVIG) for Guillain-Barre syndrome (GBS)

**Fig. 1:**
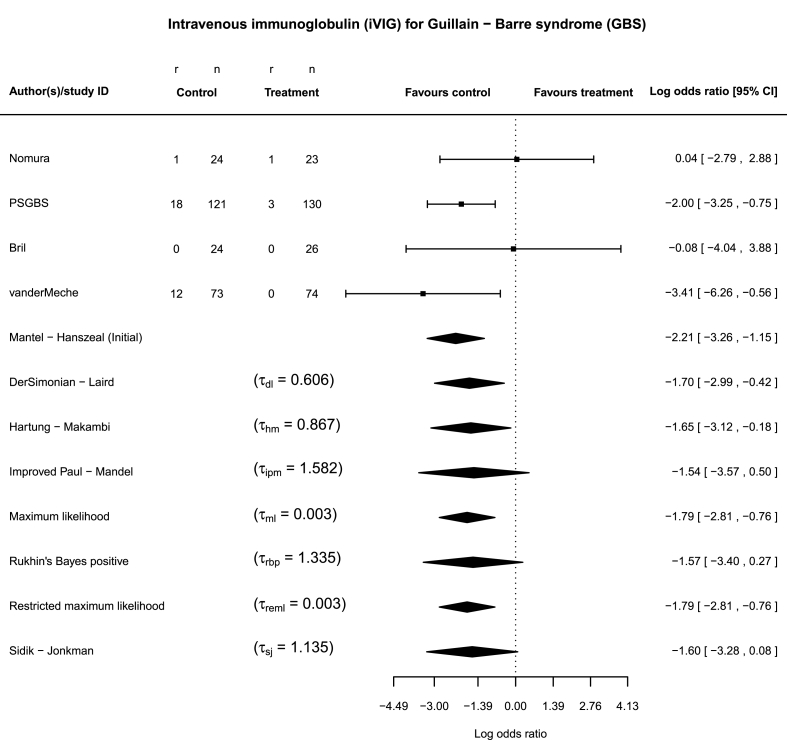
Forest plot of the overall treatment effect (log odds ratio) for the Guillain-Barre syndrome (GBS) example. The inverse-variance random-effects method is applied in combination with the seven heterogeneity estimators. The between-study standard deviations *τ* are presented alongside each estimator The confidence intervals are calculated as $\hat{\theta }\pm {\hat{\sigma }}_{\theta }\cdot Z_{1-\alpha /2}$. The Mantel-Haenszel analysis is plotted as well.

GBS syndrome has a prevalence of 1–9/100.000 [29], the term is used to describe a number of rare post-infection neuropathies. Patients may recover completely, remain unable to walk 6 months after disease onset or have a fatal outcome. A recent Cochrane review and MA summarized four RCTs that compared IVIG to plasma exchange [4]. Treatment discontinuation was reported, as a secondary outcome. Trials which were relatively small either failed to report any event or they only had one in each arm. On the contrary, the largest of these trials reported a considerable number of events in both arms (Fig. 1). For the initial analysis the Mantel-Haenszel (MH) FE risk ratio 0.14 (95% 0.05–0.36) was used. By using the MH, the authors excluded information from trials with no reported event, which resulted in a significant overall effect with moderate estimated heterogeneity.

### 3.2 Sapropterin dihydrochloride for phenylketonuria (PHK)

PHK is a common inborn error of amino acid metabolism that causes mental disability (mild to severe) to patients who are not treated properly. It is considered a rare child disorder with a prevalence of 1–5/10.000 [29]. A Cochrane review consisted of two studies on sapropterin dihydrochloride and reported on several adverse events, such as vomiting [5]. The two studies produced contradictory but not significant results overall (Supplementary material A - Table 1). Even though, the estimated heterogeneity was substantial, the studies were again pooled using a FE MH on the risk ratio 1.04 (95% 0.28–3.91) [5].

### 3.3 Analysis of motivating examples

In regards to our first example (GBS), the final conclusion is influenced considerably by the choice of the heterogeneity estimators. Estimators that lead to a larger estimate value of *τ* fail to reject the null hypothesis and therefore result in a more conservative conclusion (Fig. 1).

In the second example (PHK), the overall treatment effect changes direction, depending on the choice of estimator (Supplementary material A - Table 2). The overall treatment effect remains non-significant due to the contradictory results of the two available trials. When estimating the heterogeneity, we observe a behaviour similar to the first example.

## 4 Simulation study

In order to assess the performance of a predefined versus a data-driven choice of analysis in the aforementioned setting, we conducted a simulation study that is divided in two parts; (1) evaluating the operational characteristics for the whole simulation, which represents the ”*unconditional approach*” strategy and (2) evaluating the operational characteristics for subsets of the whole simulation that are defined by the number of observed zero cells in a simulated MA. The second part represents the ”*conditional approach*” strategy.

### 4.1 Unconditional approach

Following the strategy of Hartung and Knapp [30] we simulated logORs from the null and alternative hypothesis. We varied the overall treatment effect as $\theta_{r}\in \left\{0,\;1\right\}$ and set the heterogeneity equal to $\tau_{r}^{2}\in \left\{0,\;0.5,\;1,\;2\right\}$. These four values correspond to $\tau_{r}\in \left\{0,\;0.71,\;1,\;1.41\right\}$ and to $I_{r}^{2}≃\left\{0\text{\%},\;40\text{\%},\;60\text{\%},\;75\text{\%}\right\}$ levels of relative heterogeneity, which are calculated via simulation of $I^{2}=\tau^{2}/\left(\tau^{2}+{\bar{s}}^{2}\right)$, ${\bar{s}}^{2}=$ $\sum_{j=1}^{10^{5}}s_{j}^{2}$ $/10^{5}$ where j: number of simulations. The total number of trials was restricted within k∈{2,3,4}. Ten fixed values as of $P_{cr}\in \left\{0.05,0.06,\ldots ,0.15\right\}$ were used for the control group event rate of the outcome. By simulating a uniformly random draw between {[20,30]} for each trial arm, we varied the samples sizes between trials, while we kept the allocation ratio within each trial equal to 1:1. The sample size and allocation ratio were kept fixed in order for similar number of zero events to be produced in each arm. In this way, the small sample sizes in combination with different levels of control event rate led to specific levels of expected zero-event arm percentages (Supplementary material A - Table 3).

### 4.2 Conditional approach

For the second approach we focused on the evaluation of a four (k = 4) trial MA, since the relative performance of the heterogeneity estimators was similar across k = 2,3,4 trials. The conditional simulation theoretically leads up to a maximum of 8 distinct subsets, since a four trial MA results from a minimum of 1 to a maximum of 8 zero-event arms. Of course, the latter ones are not useful to consider for a meta-analysis.

For the unconditional approach we based performance measures on 10,000 simulated MAs and evaluated all 15 *τ* estimators, while for the conditional approach we based performance measures on 1,000,000 simulated MAs and evaluated 7 selected *τ* estimators that we considered important from the unconditional analysis. A constant CC of 0.5 was added to all cells of a trial that reported at least one zero event. All computations were performed using R and the high performance cluster supported by Utrecht Bioinformatics Center. An overview of the varied parameters for each simulation approach is presented in Supplementary material A (Table 4).

### 4.3 Performance measures

We assessed the bias of heterogeneity and overall treatment effect estimates. We calculated the empirical type I error, the power and coverage of the 95% confidence intervals of the overall effect estimate. Finally, we computed the probability of each estimator to observe a non-zero heterogeneity estimate ($Pr\left({\hat{\tau }}^{2}\right)>0$).

## 5 Results

In our small population settings, many heterogeneity estimators performed similarly. More specifically, estimators can be grouped -based on their performance-in two groups. Estimators dl, dl2, dlp, he, he2, mvvc, pm and rb0 displayed similar behaviour in our study. Estimators ml and hs showed a similar insufficient performance in identifying heterogeneity (Supplementary material B). Based on this we selected a key set of 7 estimators for detailed evaluation; dl from the first group, ml from the second group and five estimators that displayed the most divergent behaviour sj, ipm, rbp, hm and reml. In the case of two studies, most heterogeneity estimators behaved similarly.

**Fig. 2:**
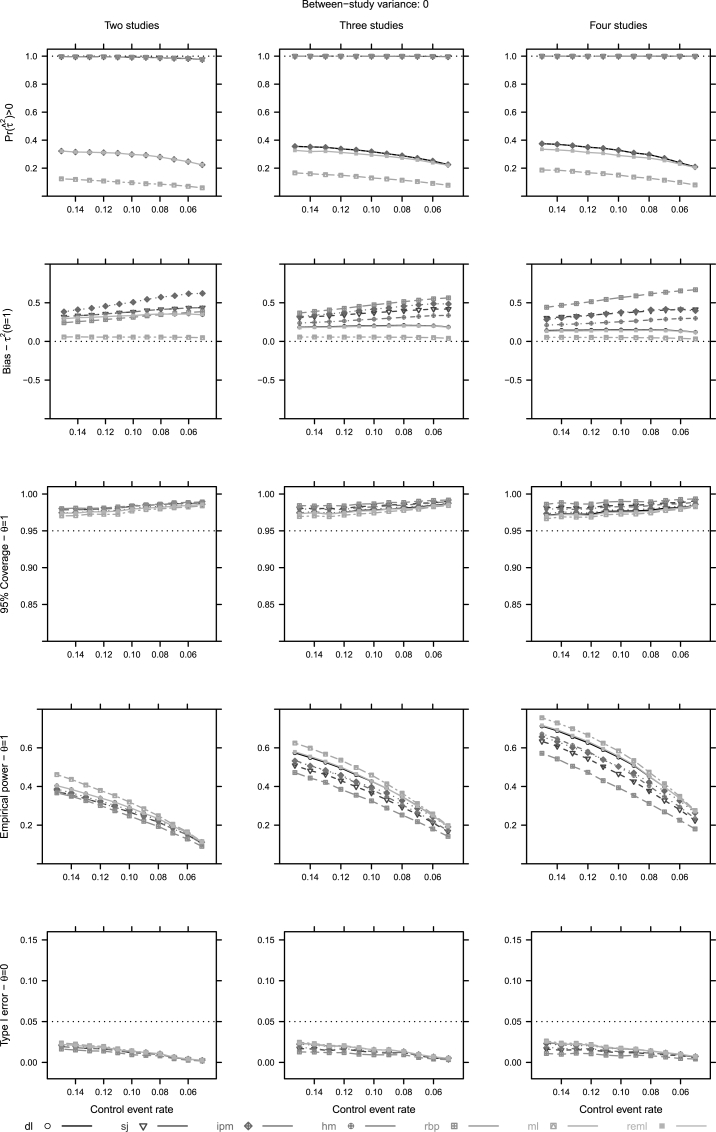
Unconditional approach operational characteristics ($Pr\left({\hat{\tau }}^{2}\right)>0$, mean bias of *τ*, coverage of the 95% confidence intervals, empirical power and type I error of *θ*) for two to four studies and $\tau_{r}^{2}=0$. For abbreviations see Table 1.

**Fig. 3:**
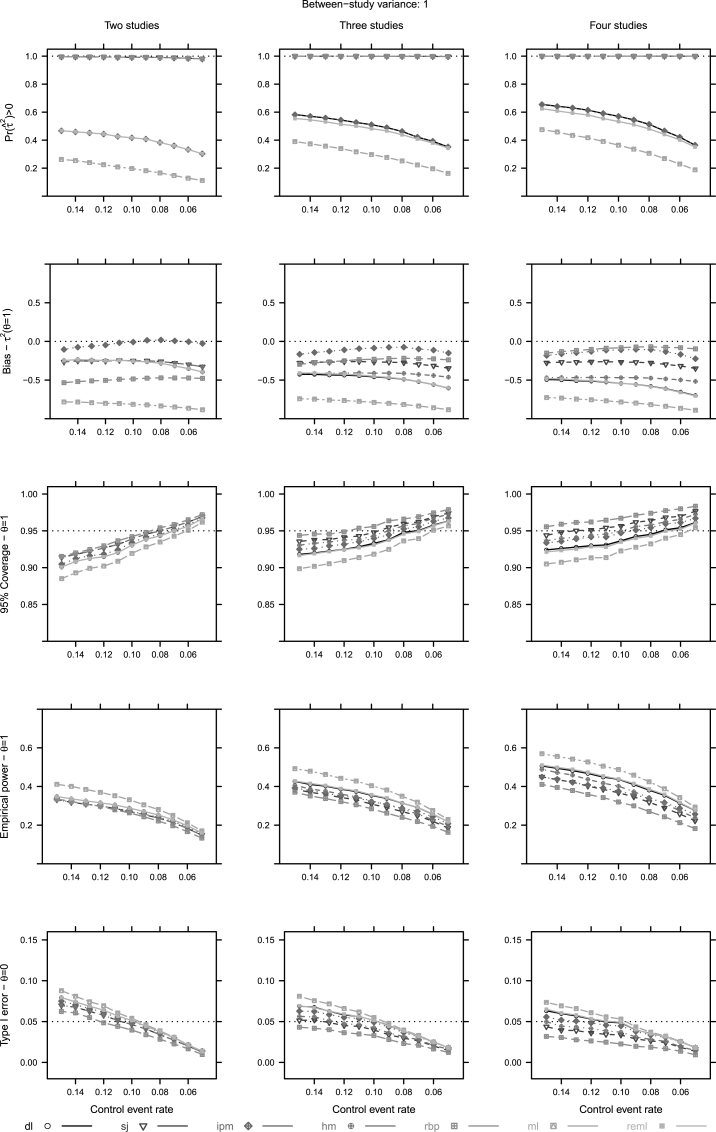
Unconditional approach operational characteristics ($Pr\left({\hat{\tau }}^{2}\right)>0$, mean bias of *τ*, coverage of the 95% confidence intervals, empirical power and type I error of *θ*) for two to four studies and $\tau_{r}^{2}=1$. For abbreviations see Table 1.

**Fig. 4:**
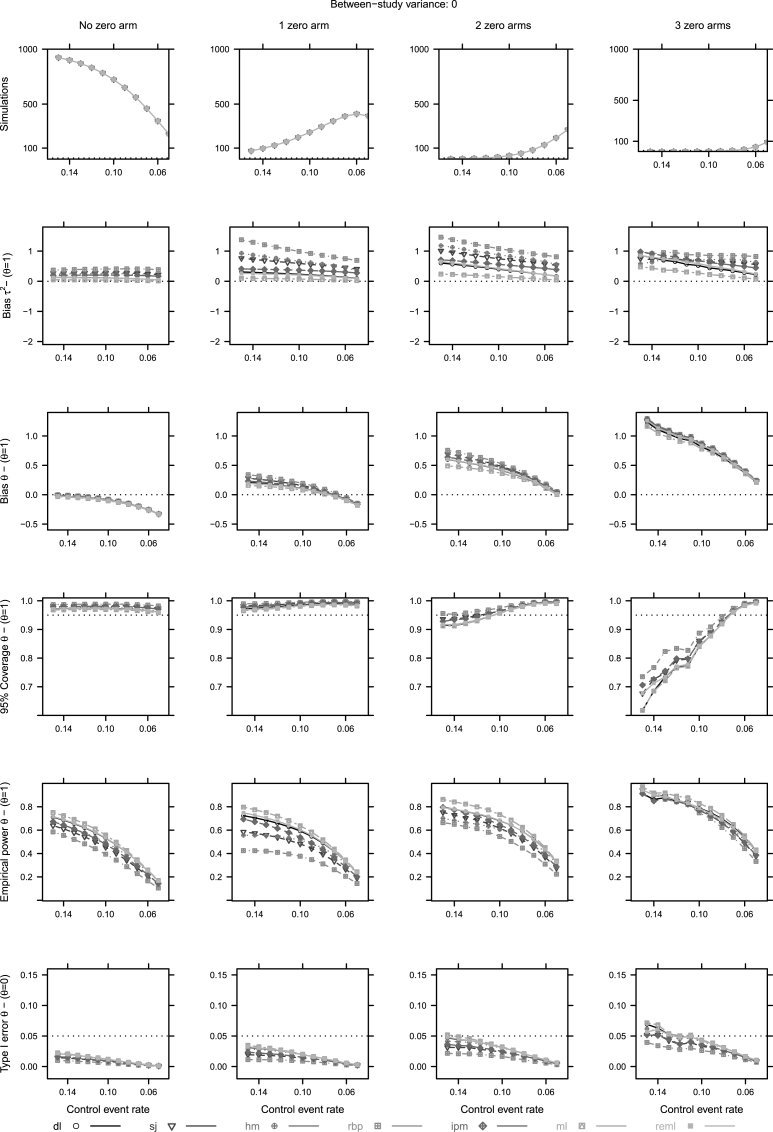
Conditional approach operational characteristics (Mean bias of *τ*, mean bias, coverage of the 95% confidence intervals, empirical power and type I error of *θ*) for four studies and $\tau_{r}^{2}=0$. For abbreviations see Table 1. First row *y*-axis - 1000: 1,000,000, 500: 500,000, 100: 100,000 simulations.

**Fig. 5:**
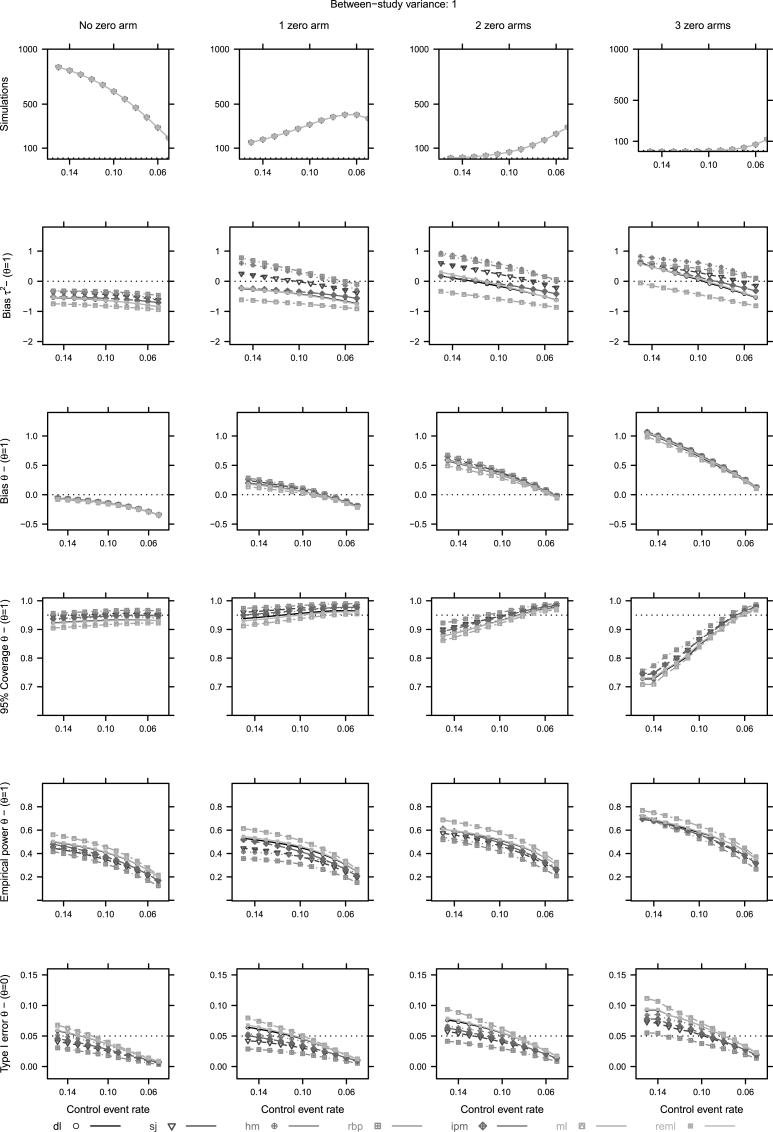
Conditional approach operational characteristics (Mean bias of *τ*, mean bias, coverage of the 95% confidence intervals, empirical power and type I error of *θ*) for four studies and $\tau^{2}=1$. For abbreviations see Table 1. First row *y*-axis - 1000: 1,000,000, 500: 500,000, 100: 100,000 simulations.

Regarding the unconditional approach, we summarize the results in two figures Fig. 2 ($\tau_{r}^{2}=0$) and Fig. 3 ($\tau_{r}^{2}=1$). The same two scenarios are presented for the conditional approach in Fig. 4, Fig. 5. Interested readers can find the figures of the remaining scenarios in Supplementary material B.

### 5.1 Unconditional approach

Alternative heterogeneity estimators had little impact on the bias of $\hat{\theta }$. As the control event rate ($P_{cr}$) decreases, bias increases for all estimators. In addition, the point estimation of *τ* is problematic as well. Under homogeneity ($\tau_{r}^{2}=0$), all estimators greatly overestimate *τ*, except for ml, while under heterogeneity ($\tau_{r}^{2}=1$) rbp, sj and ipm induce the least bias on *τ* (Fig. 2, Fig. 3).

The presence of heterogeneity impacts the type I error heavily. In non-sparse conditions, when $\tau_{r}^{2}=0$, all estimators behave conservatively in interval estimating the overall effect, while in heterogeneous conditions ($\tau_{r}^{2}=1$) most of the estimators behave liberally. On the contrary, all estimators display conservative behaviour in very sparse conditions, regardless of the presence of heterogeneity (Fig. 2, Fig. 3). In addition, decreasing $P_{cr}$ levels impact the 95% coverage. We also note that no estimator shows potential to control the coverage, when only two or three small trials are available (Fig. 2, Fig. 3).

The properties of the estimators' depend on the levels of true heterogeneity. As true heterogeneity will not be known, nor very reliably estimated we seek some robustness. And thus, we would prefer estimators that are less dependent on levels of true heterogeneity; for example, sj, hm and ipm (Fig. 2, Fig. 3).

### 5.2 Conditional approach

The first row in Fig. 4, Fig. 5 represents simulations that produce a specific number of zero cells. The first column refers to MAs with no observed zero cell. The rest refer to MAs with an exact number of observed zero cells.

In terms of bias of $\hat{\theta }$, we notice similar properties across conditional subsets; hence, an increase of negative bias, as the $P_{cr}$ decreases (Fig. 4, Fig. 5). In the particular case of exactly no zero cell we observe an overall negative bias (Fig. 4, Fig. 5). The point estimation of *τ* is impacted by zero cells as well. When no zero cell trial is observed in a MA, all estimators produce values that are relatively close to each other. The increasing number of zero cells makes the estimation of heterogeneity unstable (Fig. 4, Fig. 5).

The performance of the estimators in terms of 95% coverage and type I error, depends again on the levels of true heterogeneity. In homogeneous cases ($\tau_{r}^{2}=0$), independently of observed zero cells, all estimators lead to conservative inferences. When no zero cell trial is observed in a MA, and heterogeneity exists ($\tau_{r}^{2}=1$), then most estimators result in liberal inferences. As the number of zero cells increases, estimators result in conservative inferences (Fig. 5). Again estimators whose performance is less dependent on levels of true heterogeneity are sj, hm and ipm. In addition, ipm produces relative higher power in comparison to sj and hm when one or two zero cells are observed in a MA (Fig. 4, Fig. 5).

In the case of no observed zero cells in a MA of heterogeneous settings ($\tau_{r}^{2}\geq 1$), all estimators induce negative bias on the estimation of *θ* and the estimation of *τ* (Fig. 5). When at least one zero trial is observed, inference becomes unstable. Such a behaviour could be explained by the impact of CCs on the study weights. When a zero cell trial is observed and a CC is applied, this trial's weight decreases. Therefore, RCTs with low event rates that probably point towards a small or no treatment effect would be down-weighted.

### 5.3 Revisiting the motivating examples

According to our simulation study, the conditional selection of heterogeneity estimator, which is based on the exact number of zero cells, would bring no added value, compared to the unconditional selection of an estimator when a sparse-events MA in small populations is expected. As heterogeneity cannot be reliably estimated in such sparse settings, the chosen estimator should be robust against the level of true heterogeneity. For example, if we had selected the sj, an estimator that was found to be less impacted by the levels of true heterogeneity, we would not have rejected the null hypothesis for the GBS example (Fig. 1).

Supplementary material A (Table 2) presents an extensive analysis that demonstrates the effect of applying alternative heterogeneity estimators on the overall treatment effect for the two motivating examples.

## 6 Discussion

In this paper we assess and discuss the problematic (interval) estimation of the overall treatment effect, in the presence of heterogeneity for a MA of a few small RCTs with zero events. In this context a truly robust estimation of heterogeneity appears not feasible. Neither can we recommend a single heterogeneity estimator which provides overall satisfactory performance in our small population sparse-event setting. In addition, the comparison between the two simulation approaches showed that the relative performance of heterogeneity estimators did not differ. Therefore, there is no material issue between making a predefined (unconditional) or a data-driven (conditional) choice. Further insights are provided by the conditional approach, which showed that even one observed zero cell has a considerable impact on the inference.

When performing a MA of rare diseases with anticipated or reported zero cells, regardless of a predefined or a data-driven analysis choice, one should avoid methods whose performance depends strongly on the presence of heterogeneity. Following this context, we identify and suggest estimators that perform modestly robust in (interval) estimating the overall treatment effect across a range of heterogeneity assumptions such as sj, hm and ipm. On the contrary, estimators whose performance depends heavily on the true level of heterogeneity, such as rbp and ml, should be avoided. In such a setting, one strategy might be to apply the key set of heterogeneity estimators. If this leads to treatment effect estimates and confidence intervals, which are not comfortably in the same direction, we should probably be cautious to draw firm conclusions.

With few events, the estimated study effects are biased, a bias which reveals itself in between-study variance. Few events also result in large within-study variance which masks between-study variance. Therefore, a trade-off exists; due to the biased effect estimates, heterogeneity increases but due to the large within-study variances, heterogeneity decreases. Hence, we conclude the following; (i) when no heterogeneity exists it can only be overestimated due to the biased estimates but (ii) when large heterogeneity exists, it is masked and underestimated.

The simulation study results pair with previous research. In our small population setting, a number of heterogeneity estimators showed small differences in performance [31]. In the particular case of two studies, most of the heterogeneity estimators behaved similarly as also was theoretically expected [31]. As noted already, a considerable difference was observed on the (interval) estimation of the overall treatment effect among heterogeneity estimators that are known to overestimate (rbp) or underestimate (ml) the true heterogeneity [17,25,28]. Such choices should be avoided in our setting as their performance is dependent on the level of true heterogeneity, which cannot be properly estimated. Furthermore, note that simulation studies results in such settings can significantly depend on the Data-Generating Model employed in the design of the simulation [32]. The relative behaviour of the compared heterogeneity estimators did not differ under alternative generating models applied. Relative behaviour also did not differ when applying a CC of 0.1 instead of 0.5, though the general performance of the inverse variance method was affected (see Supplementary Material B).

We only considered a simple Wald test for hypothesis testing via the IV method. We note the existence of an alternative test [30], which has the ability to control the type I error, in a more effective manner than the Wald test for a small number of trials. However, this test does not have sufficient power to detect a true effect [33,34]. In addition, the simple IV RE model might underperform in a few trials MA, thus sophisticated techniques that control type I error might be preferred. In this context a sensitivity analysis based on a variety of techniques was suggested [35].

We also restricted our comparison to the commonly employed RE model which assumes normally distributed effects across studies, with a common variance. This assumption and Data Generating Model has been challenged, and alternative methods for data synthesis, based on quasi-likelihood approaches have been proposed [36]. Such methods might be useful for robust interval estimation but their operational characteristics need to be further examined.

Simulation studies have evaluated several other meta-analytical methods regarding their ability to account for zero cells [9,[12], [13], [14]]. Among others, they include: (1) the evaluated IV method with alternative CCs [9], (2) the Peto method, which excludes trials with zero events in both arms internally from a MA [14], (3) the MH method for the OR [14], (4) methods that use alternative effect measures, such as the arcsine difference [13] and (5) multilevel models or with alternations in their likelihood [12]. The latter are prone to convergence issues when the number of levels (groups or trials) and the number of events or patients is limited [12,37]. These studies [9,[12], [13], [14]] focused on sparse-events MA, particularly in cases of relatively large sample sizes and large numbers of available studies. Hence, results could not be generalized directly to rare diseases, as the latter have both a limited number of trials and small sample sizes. Further research could focus on the aforementioned methods' behaviour, on the basis of the exact number of observed zero cells in a MA when only a few trials are available.

Further, by utilizing historical data, experts' opinions or priors that cover plausible heterogeneity values, Bayesian inference might provide a suitable alternative for cases of small populations [[38], [39], [40]]. Although it was not our primary focus, initial evaluations showed that a similar two-level normal Bayesian hierarchical model combined with informative priors [39] produces smaller biases on the estimation of heterogeneity but similarly problematic 95% coverage for very low control event rates.

In this study, we did not evaluate heterogeneity estimation within complex meta-analytical settings, such as a multiple outcome MA [41] or a network MA [42,43]. However, we expect that the impact of zero cells in small MAs could be relevant for this context as well, and a similar conditional examination could offer further insight.

Concluding, the choice of heterogeneity estimator does not need to depend on whether or not zero cells are observed in a MA of few small trials. Therefore, regardless of a predefined or data-driven analysis choice, when dealing with zero cells in a MA of rare diseases, we recommend methods with performance that does not strongly depend on the presence or absence of heterogeneity.

### Author's contributions

KP, SN, DM and KR contributed to the conception and design of the study. KP performed the simulation study and produced the first draft. SN, KR and DM critically reviewed the article. All authors have read and accepted the final manuscript.

## Appendix A Supplementary data

mmc1 mmc1

mmc2 mmc2

mmc3 mmc3
