# Supplementary material for: Interval estimation of the overall treatment effect in a meta-analysis of a few small studies with zero events
Source: Contemp Clin Trials Commun. 2018 Jan 9;9:98–107. doi: 10.1016/j.conctc.2017.11.012 (PMC5898531; doi:10.1016/j.conctc.2017.11.012)
Supplement: mmc2 [file mmc2.pdf]

Supplementary material B

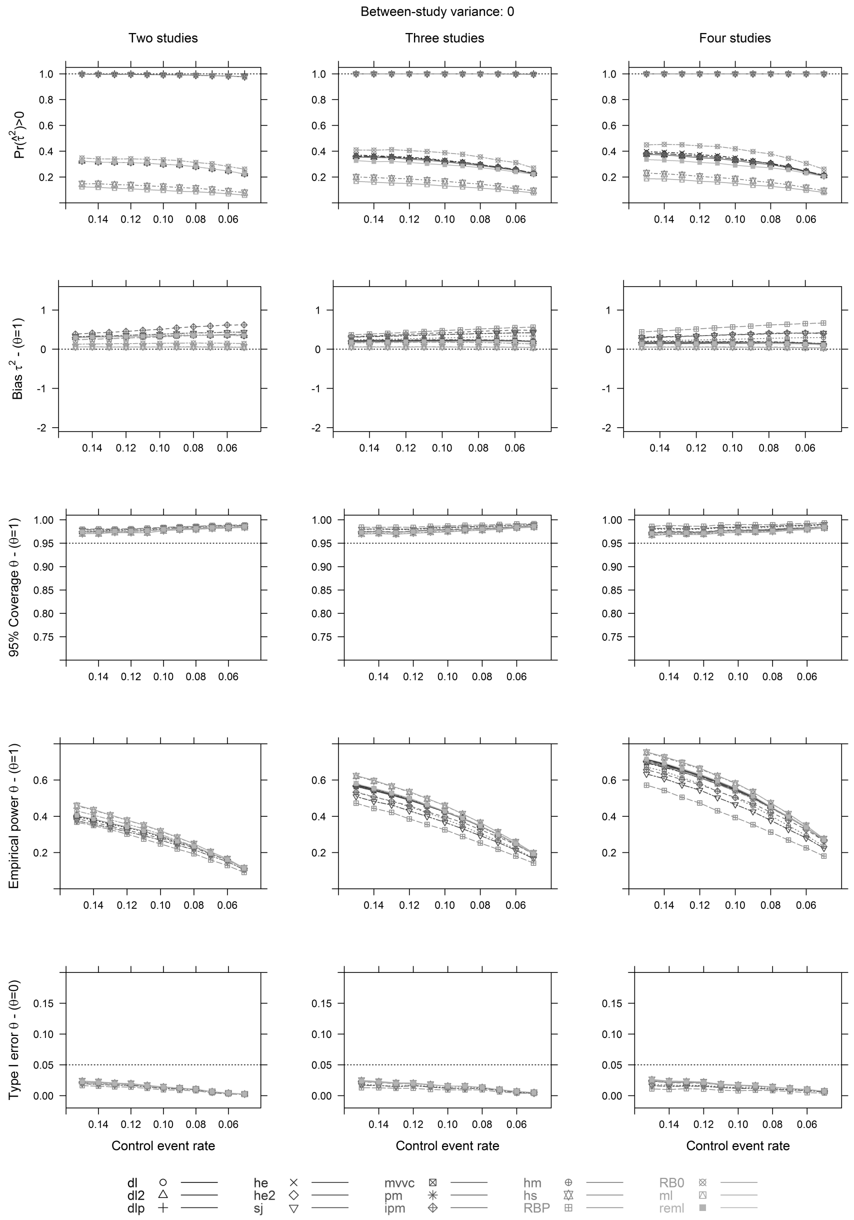

Figure 1: Main simulation scheme operational characteristics ($Pr(\hat{\tau}) > 0$, mean bias of τ, 95% coverage probability, empirical power and empirical type I error of θ) for a selected scenario of two to four studies. For abbreviations see Table 1 (Main manuscript).

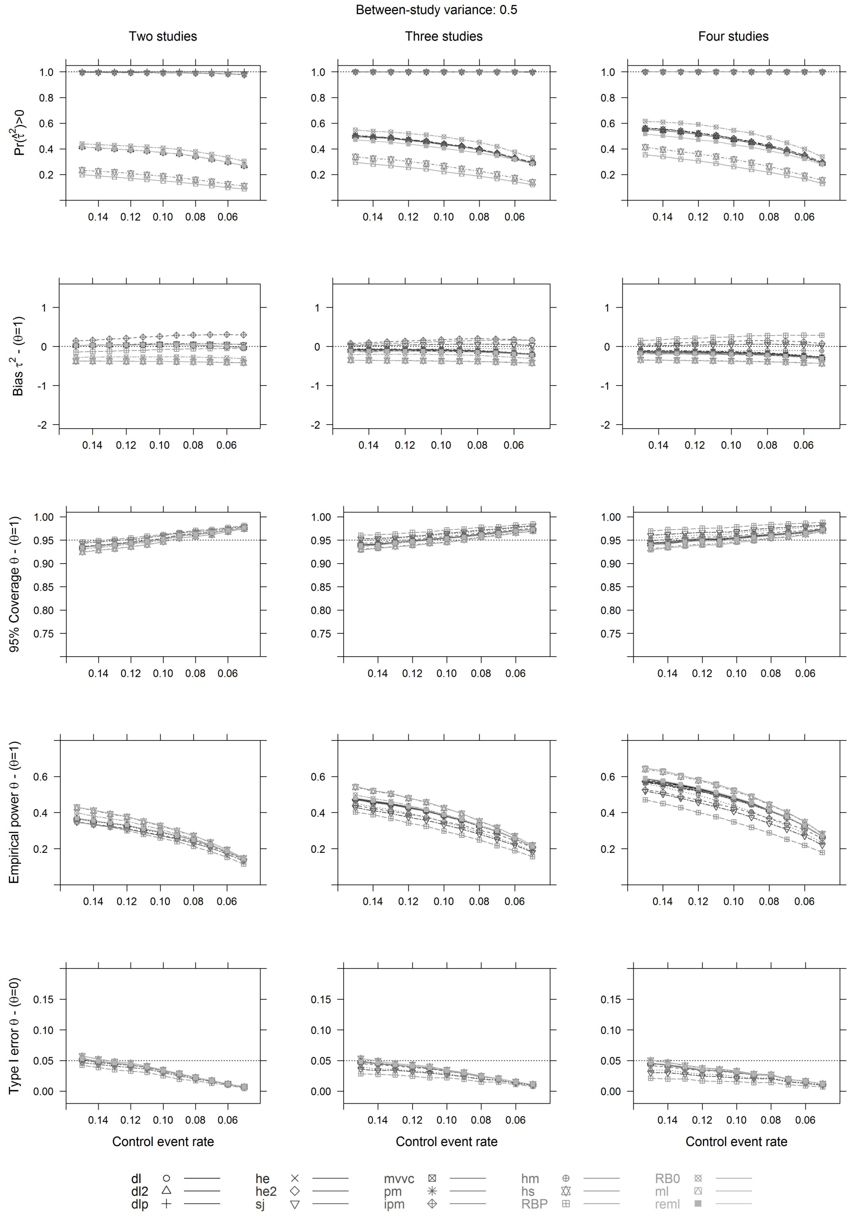

Figure 2: Main simulation scheme operational characteristics ($Pr(\hat{\tau}) > 0$, mean bias of τ, 95% coverage probability, empirical power and empirical type I error of θ) for a selected scenario of two to four studies. For abbreviations see Table 1 (Main manuscript).

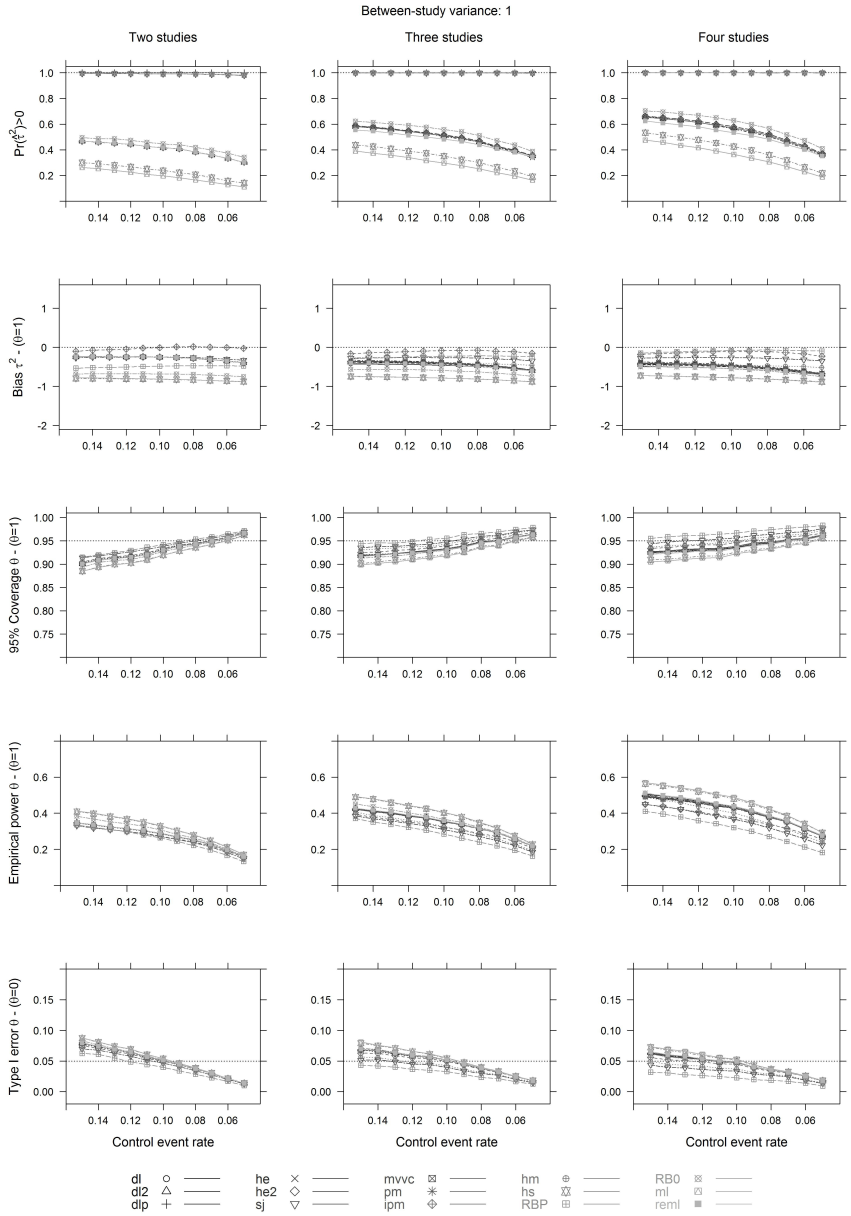

Figure 3: Main simulation scheme operational characteristics ($Pr(\hat{\tau}) > 0$, mean bias of τ, 95% coverage probability, empirical power and empirical type I error of θ) for a selected scenario of two to four studies. For abbreviations see Table 1 (Main manuscript).

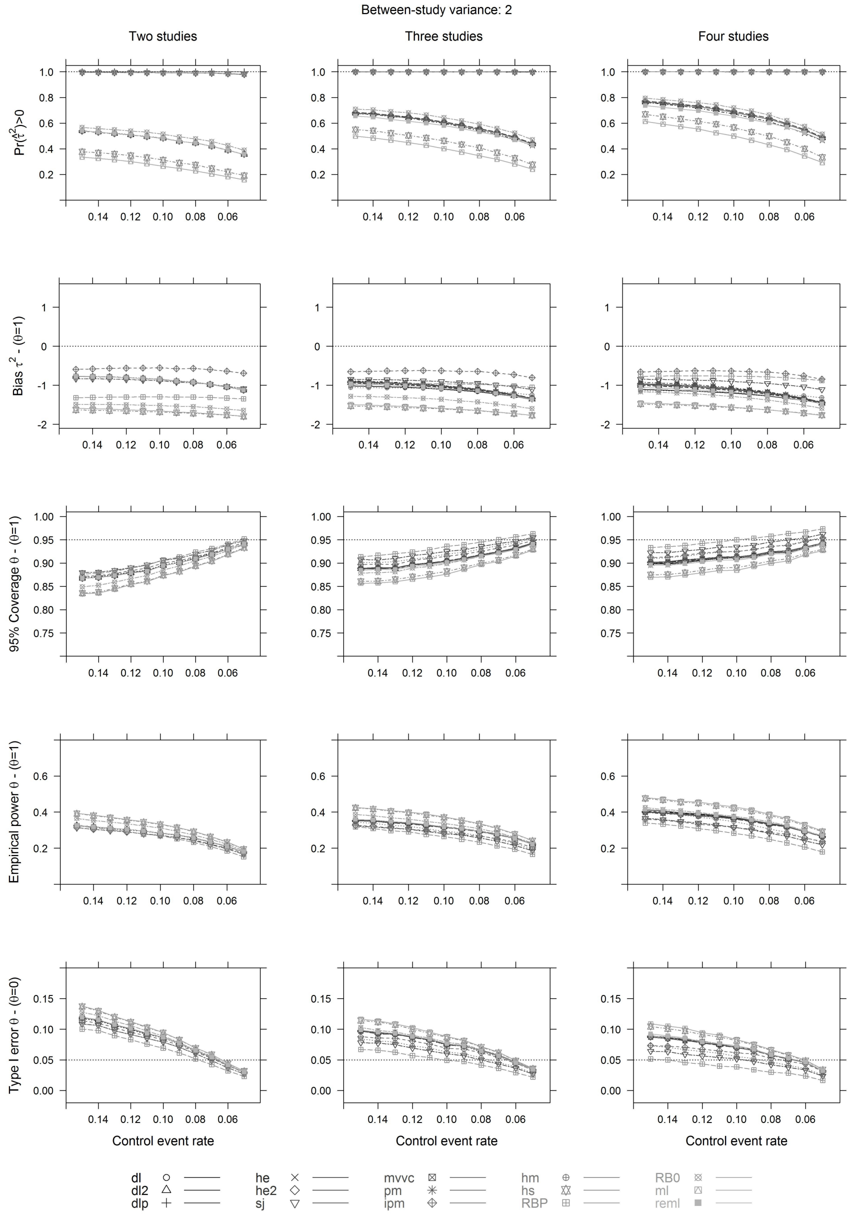

Figure 4: Main simulation scheme operational characteristics ($Pr(\hat{\tau}) > 0$, mean bias of τ, 95% coverage probability, empirical power and empirical type I error of θ) for a selected scenario of two to four studies. For abbreviations see Table 1 (Main manuscript).

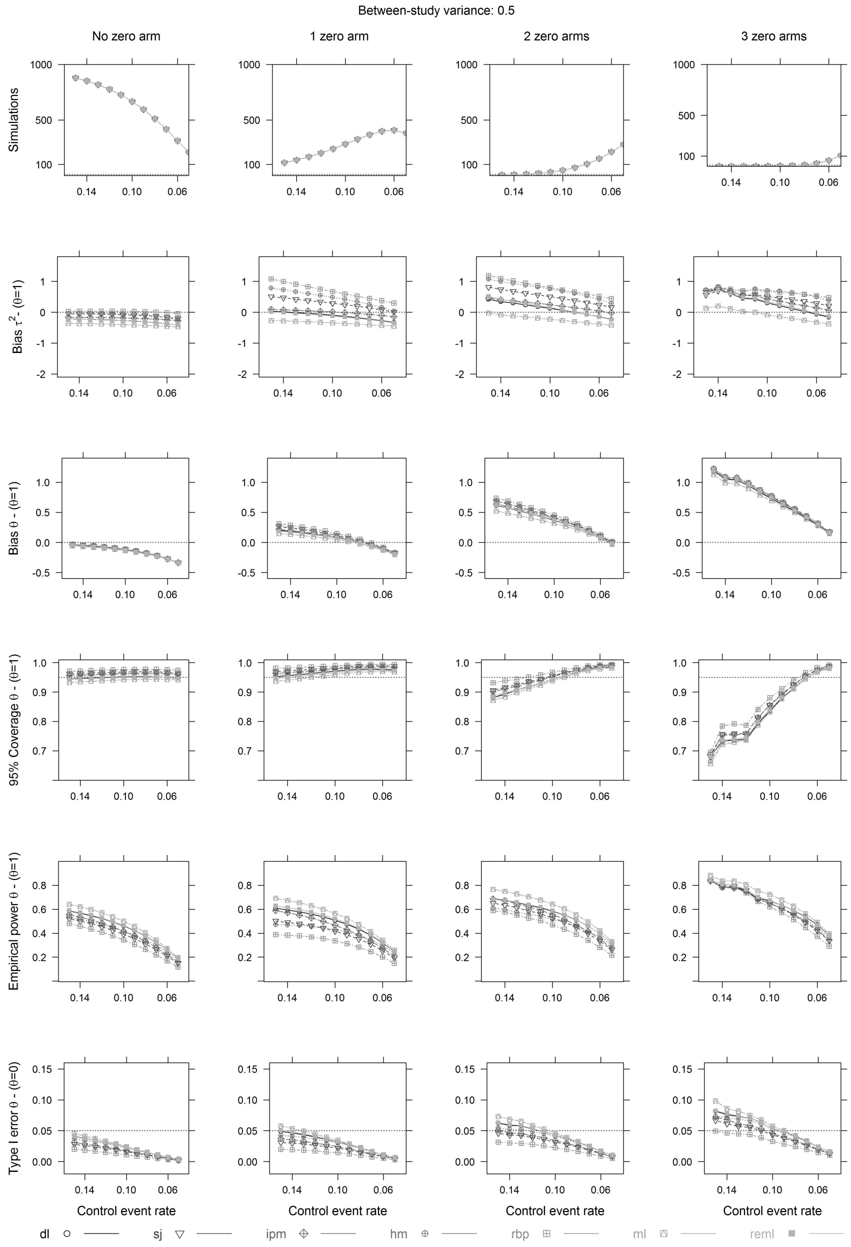

Figure 5: Conditional subset simulation scheme operational characteristics (Mean bias of τ, mean bias, 95% coverage probability, empirical power and empirical type I error of θ) for four studies and $\tau^2 = 0.5$. For abbreviations see Table 1 (Main manuscript). First row y-axis - 100: 100,000, 50: 50,000, 10: 10,000 simulations

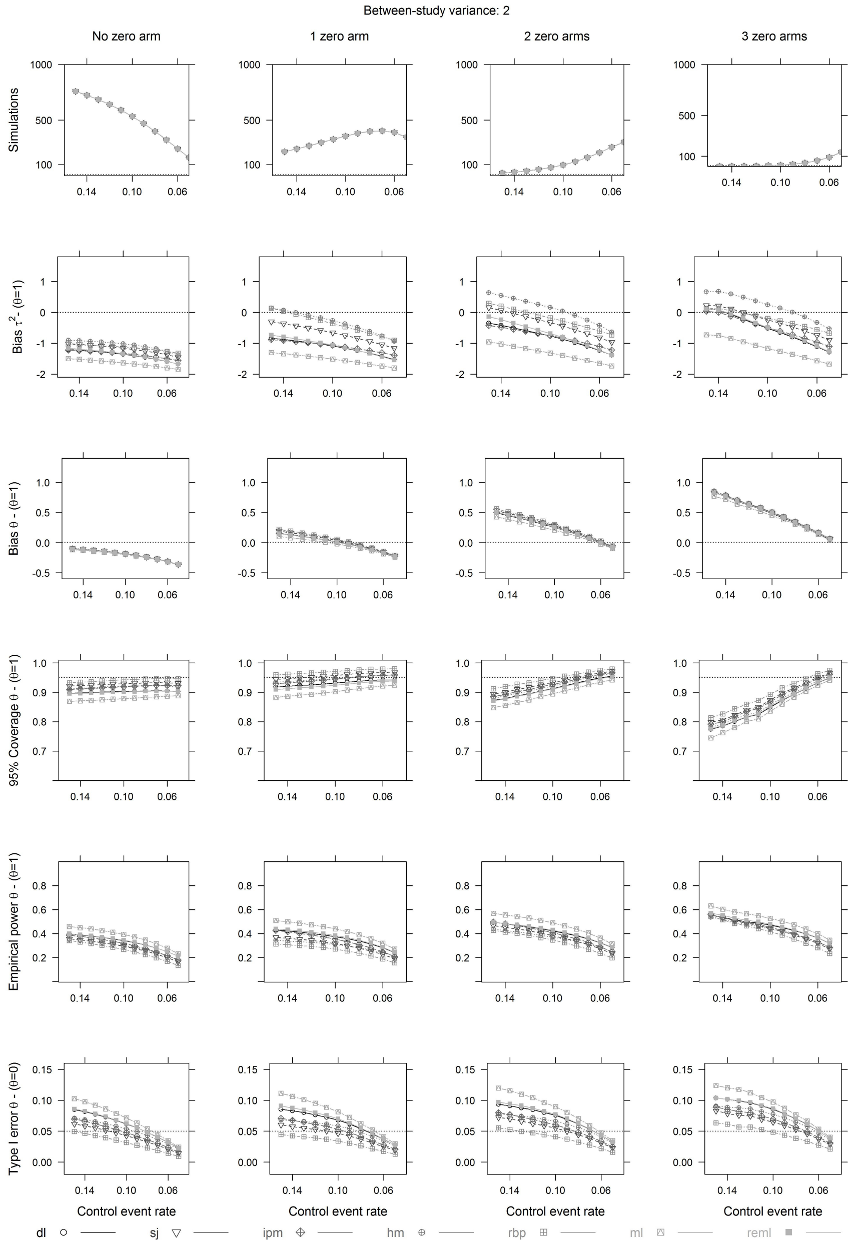

Figure 6: Conditional subset simulation scheme operational characteristics (Mean bias of τ, mean bias, 95% coverage probability, empirical power and empirical type I error of θ) for four studies and $\tau^2 = 2$. For abbreviations see Table 1 (Main manuscript). First row y-axis - 100: 100,000, 50: 50,000, 10: 10,000 simulations

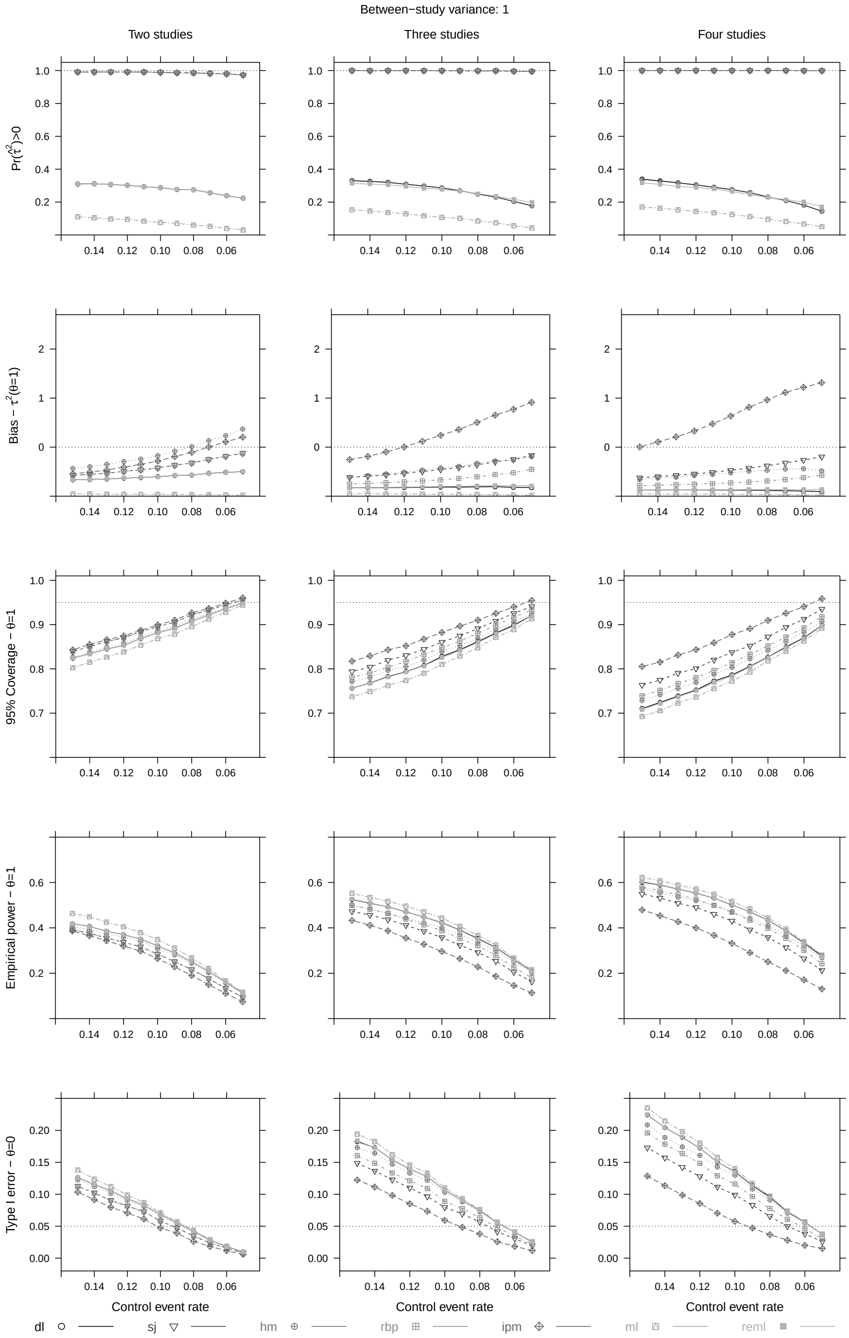

Figure 7: Main simulation scheme operational characteristics ($Pr(\hat{\tau}) > 0$, mean bias of τ, 95% coverage probability, empirical power and empirical type I error of θ) for a selected scenario of two to four studies with a constant continuity correction of 0.1. For abbreviations see Table 1 (Main manuscript).

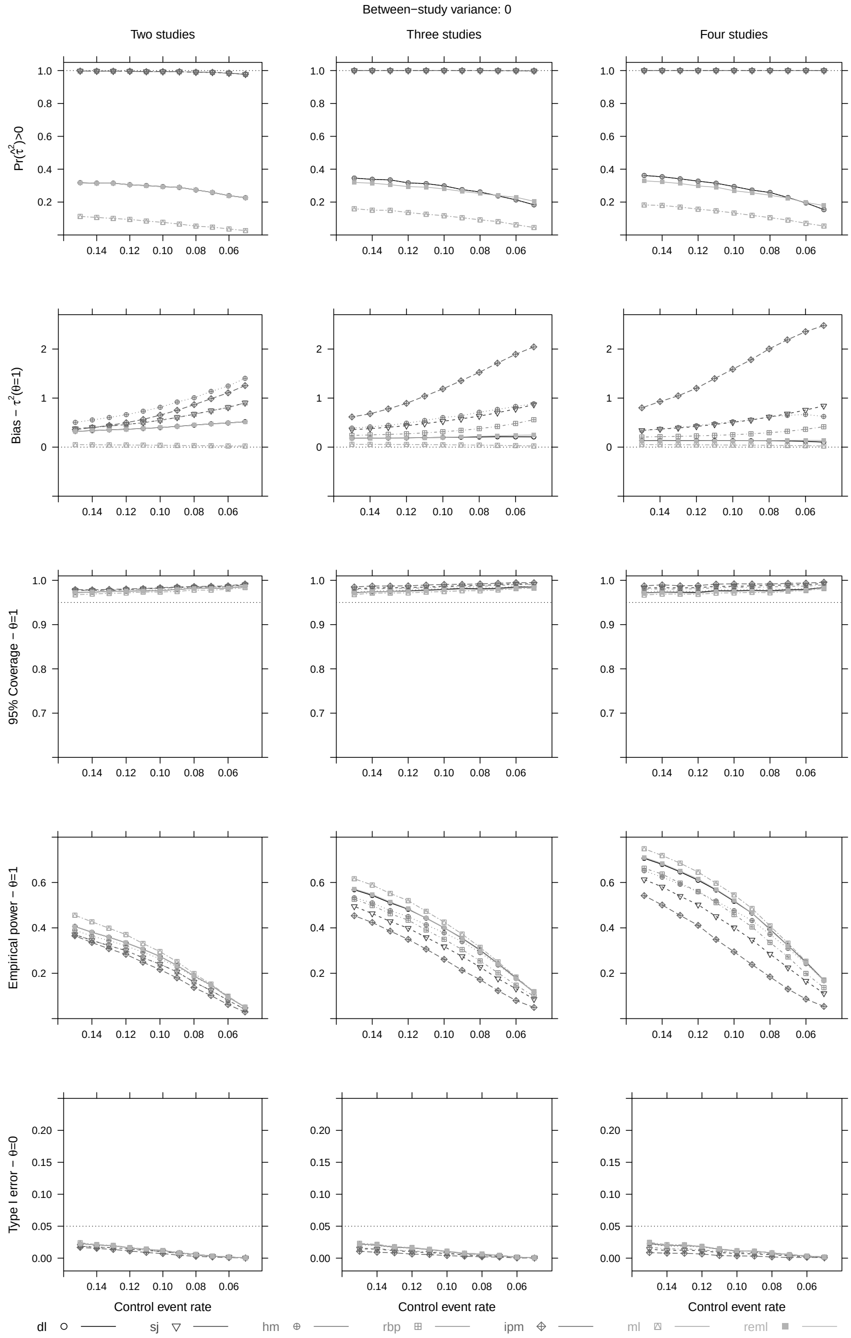

Figure 8: Main simulation scheme operational characteristics ($Pr(\hat{\tau}) > 0$, mean bias of τ, 95% coverage probability, empirical power and empirical type I error of θ) for a selected scenario of two to four studies with a constant continuity correction of 0.1. For abbreviations see Table 1 (Main manuscript).

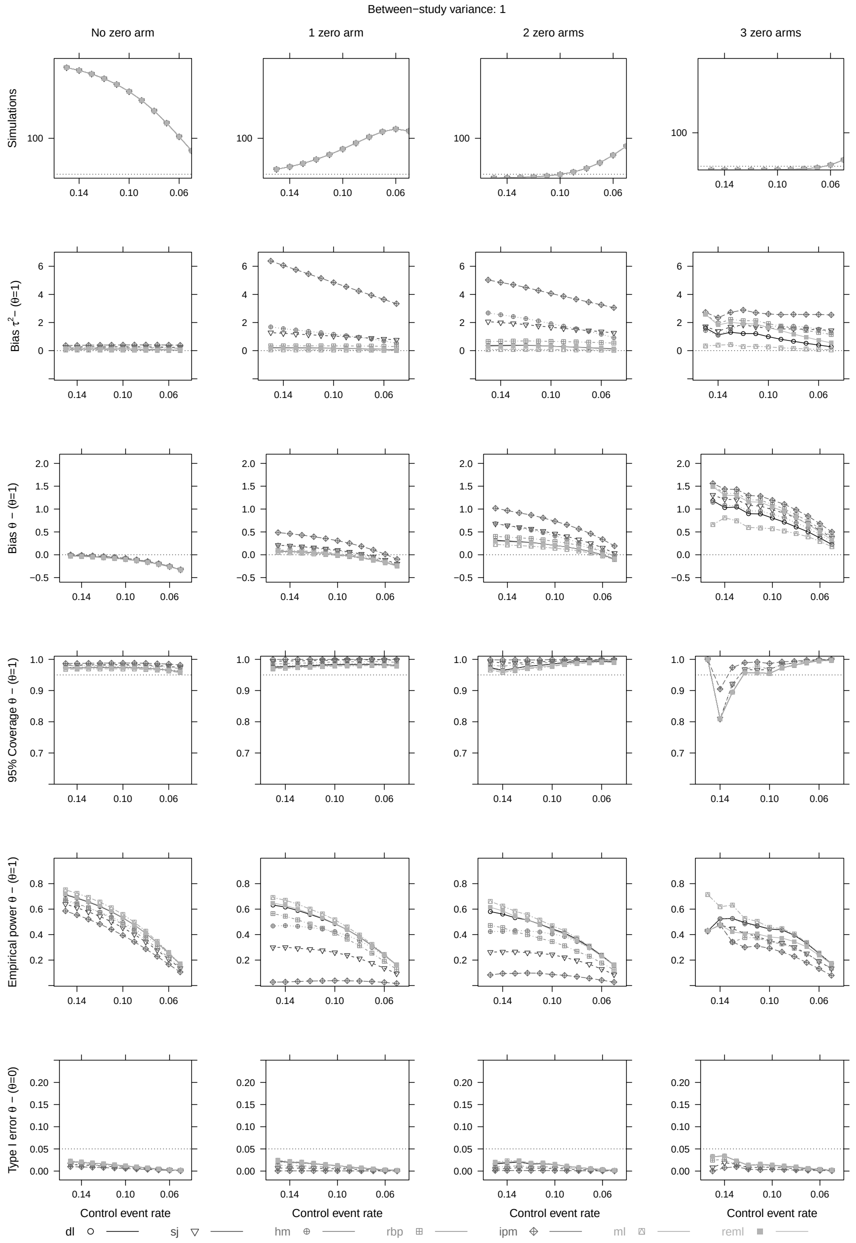

Figure 9: Conditional subset simulation scheme operational characteristics (Mean bias of τ, mean bias, 95% coverage probability, empirical power and empirical type I error of θ) for four studies and $\tau^2 = 2$ with a constant continuity correction of 0.1.. For abbreviations see Table 1 (Main manuscript). First row y-axis - 100: 100,000, 50: 50,000, 10: 10,000 simulations

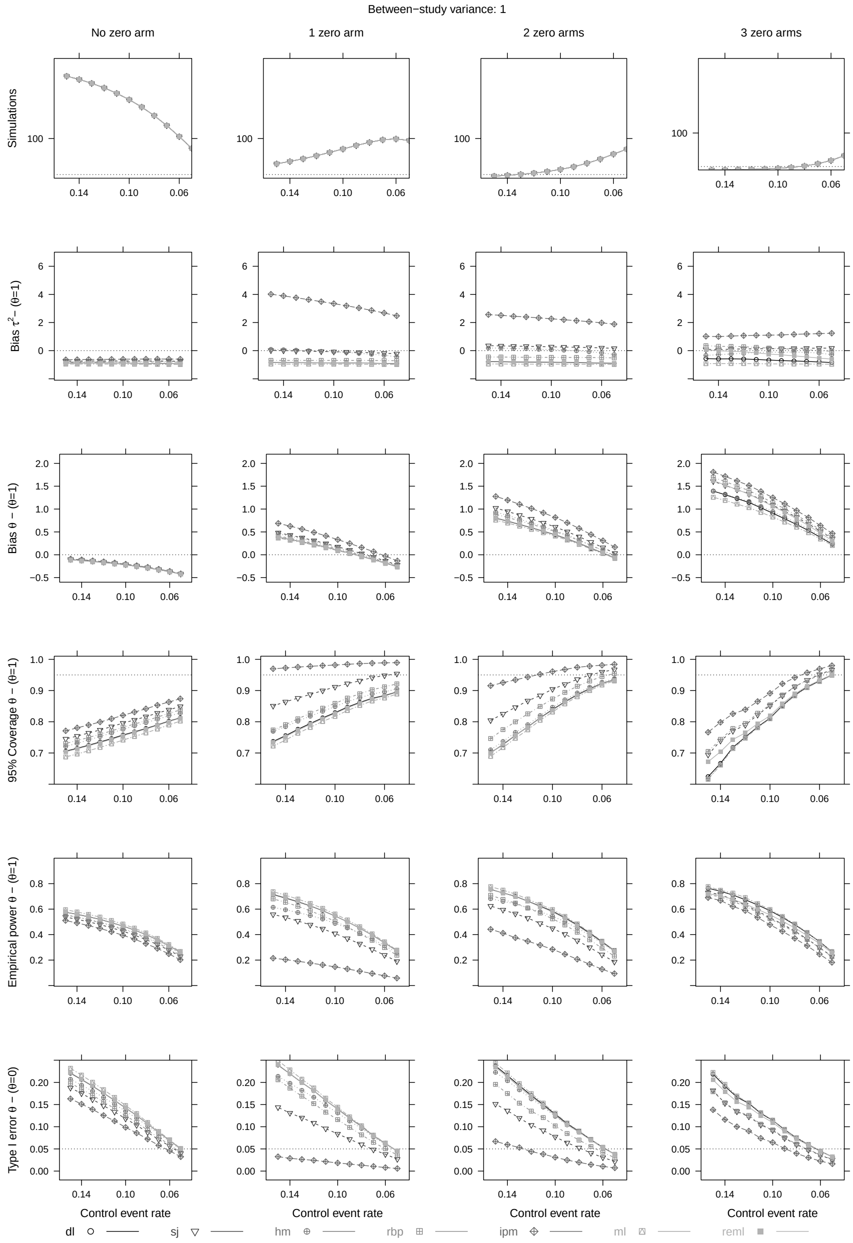

Figure 10: Conditional subset simulation scheme operational characteristics (Mean bias of τ, mean bias, 95% coverage probability, empirical power and empirical type I error of θ) for four studies and $\tau^2 = 2$ with a constant continuity correction of 0.1.. For abbreviations see Table 1 (Main manuscript). First row y-axis - 100: 100,000, 50: 50,000, 10: 10,000 simulations
