# Supplementary material for: Interval estimation of the overall treatment effect in a meta-analysis of a few small studies with zero events
Source: Contemp Clin Trials Commun. 2018 Jan 9;9:98–107. doi: 10.1016/j.conctc.2017.11.012 (PMC5898531; doi:10.1016/j.conctc.2017.11.012)
Supplement: mmc3 [file mmc3.pdf]

Supplementary material A

Table 1: **(1)** Efficacy endpoint : Treatment discontinuation, Therapy : Intravenous immunoglobulin vs Plasma Exchange, Condition : Guillain-Barre syndrome **(2)** Safety endpoint : Vomiting, Therapy : Sapropterin dihydrochloride , Condition : Phenylketonuria, P_{event} : Observed probability of event in each trial, $W_{initial}$: Weight of original analysis

Author	Year	r_{Ti}	$n_{Ti} - r_{Ti}$	r_{Ci}	$n_{Ci} - r_{Ci}$	n_{Ti}	n_{Ci}	P_{event}	$W_{initial}$
**(1)** Guillain-Barre syndrome - Treatment discontinuation									
vanderMeche	1992	0	74	12	61	74	73	0.08	0.39
Bril	1996	0	26	0	24	26	24	0	0
PSGBS	1997	3	127	18	103	130	121	0.09	0.58
Nomura	2001	1	22	1	23	23	24	0.04	0.03
**(2)** Phenylketonuria - Vomiting									
Levy	2007	2	39	4	43	41	47	0.07	0.79
Trefz	2009	4	29	0	12	33	12	0.09	0.21

Table 2: Analysis of motivating examples. This table presents a treatment effect estimate $\theta = log(OR)$, a between-study variance estimate τ^2 and the corresponding significance level (pv_θ) of a Wald test on θ. LRR: Log Relative Risk, I^2: relative heterogeneity.

Characteristics / Studies	GBS (Hughes 2014)			PHK (Somaraju 2015)		
Number of Studies		4			2	
Total Sample		495			133	
Number of Single Zeros		2			1	
Number of Double Zeros		1			0	
Init. Analysis (LRR, I^2, pv_θ)	-1.96	32%	<0.001	0.04	44%	0.69
	θ	τ^2	pv_θ	θ	τ^2	pv_θ
Hunter-Schmidt	-1.79	0	<0.01	-0.10	0	0.89
Maximum likelihood	-1.79	<0.01	<0.01	-0.10	<0.01	0.89
Restricted maximum likelihood	-1.79	<0.01	<0.01	-0.03	0.30	0.98
DerSimonian Laird (dl)	-1.70	0.37	0.01	-0.03	0.30	0.98
Two step dl	-1.69	0.46	0.01	-0.03	0.30	0.98
Positive dl	-1.69	0.46	0.01	-0.03	0.30	0.98
Hedges	-1.67	0.57	0.02	-0.03	0.30	0.98
Two step Hedges	-1.68	0.49	0.01	-0.03	0.30	0.98
Model error Variance	-1.67	0.55	0.02	-0.03	0.30	0.98
Paule Mandel	-1.68	0.47	0.01	-0.03	0.30	0.98
Rukhin's Bayes zero	-1.68	0.52	0.02	-0.03	0.29	0.97
Hartung-Makambi	-1.65	0.75	0.03	0.04	0.70	0.96
Rukhin Bayes positive	-1.57	1.78	0.09	0.05	0.77	0.96
Positive Sidik & Jonkman	-1.60	1.29	0.06	0.04	0.70	0.96
Improved Paule-Mandel	-1.54	2.50	0.14	0.14	1.58	0.91

Table 3: Calibration of probability for observing a trial with at least one zero cell (SZ), with both zero cells (DZ), all trials with at least one zero cell (All SZ), all trials with both zero arms (All DZ) in a MA in (%) under combinations of control group event rates (P_c) and treatment effects $\theta = log(OR)$ for 2 to 4 studies of 20 to 30 patients per trial arm and low heterogeneity.

		# studies :	2-4	2-4	2	3	4	2	3	4
P_c	θ	Sample $U(\alpha, \beta)$	*SZ*	*DZ*	*All SZ*			*All DZ*		
0.05	0	(20-30)	48.59	7.91	16.24	5.16	1.57	0.65	0.07	0
0.10	0	(20-30)	15.25	0.60	1.45	0.18	0.02	0	0	0
0.15	0	(20-30)	4.50	0.03	0.11	0	0	0	0	0
0.05	1	(20-30)	31.08	1.18	8.45	2.39	0.81	0.02	0	0
0.10	1	(20-30)	8.06	0.02	0.66	0.09	0.01	0	0	0
0.15	1	(20-30)	2.25	0	0.04	0	0	0	0	0

Table 4: Scenarios considered in the simulation study.

Parameters	Prospective approach	Retrospective approach
Number of trials (k)	2, 3, 4	4
Overall treatment effect (θ)	0, 1	0, 1
Heterogeneity (τ^2)	0, 0.5, 1, 2	0, 0.5, 1, 2
Control event rate (P_c)	0.05, 0.06, ..., 0.15	0.05, 0.06, ..., 0.15
Trial arm sample size ($n_{Ci} = n_{Ti}$)	(20, 30)	(20, 30)
Methods ($\hat{\tau}$)	Fifteen (15)	Seven (7)
